# Supplementary material for: Frailty predicts surgical complications after kidney transplantation. A propensity score matched study
Source: PLoS One. 2020 Feb 26;15(2):e0229531. doi: 10.1371/journal.pone.0229531 (PMC7043931; doi:10.1371/journal.pone.0229531)
Supplement: S1 Appendix — (PDF) [file pone.0229531.s001.pdf]

**S1 Appendix - Surgical protocol, prophylaxis against surgical site infections, and prevention of vascular complications in the kidney transplant unit.**

All kidney transplantations (KTx) were performed in a single institution by the same surgical team made up of six surgeons with recognized expertise in this field. The surgical technique employed was the same for all cases, using the classic technique of kidney transplant surgery.

Briefly, the organ was extraperitoneally placed in the iliac fossa contralateral to the transplanted kidney through a Gibson incision. In men, the spermatic cord was mobilized and preserved. After opening the peritoneum, the external iliac vein and artery were dissected, and the adjacent lymphatic vessels were carefully tied with 4.0 cotton suture. The renal vein was anastomosed termino-laterally to the external iliac vein with a 5.0 polypropylene suture, in continuous fashion, and the renal artery was sutured to the external iliac artery with 6.0 polypropylene suture, as described for the renal vein. For ureteral implantation, the extravesical ureteroneocystostomy was used, according to the technique proposed by Lich-Gregoir,[1,2] with modifications. Following thorough local hemostasis, the abdominal wall was closed by planes after placing the suction drain adjacent to the transplanted kidney. Both the drain and the Foley catheter were removed on the fifth postoperative day.

To minimize the occurrence of infectious complications related to the surgical procedure, one gram of first-generation cephalosporin (cefazolin) was administered intravenously during anesthetic induction. A double-J ureteral

catheter was left for 21 days in all KTx procedures performed with kidneys from a deceased donor, according to the unit's protocol.

In cases where it was necessary to use vascular microsurgery (double or triple arteries) and/or surgical stretching of the vein (right kidney), prophylaxis of vascular thrombosis was started immediately with the administration of 5,000 U of heparin, subcutaneously, every eight hours. On the following day, acetylsalicylic acid (ASA) was administered orally at a dose of 100 mg/day. Heparin was discontinued on the seventh postoperative day, and ASA was maintained for 30 days after surgery.

**References**

1. Lich RJr, Howerton LW, Davis LA. Recurrent urosepsis in children. J Urol. 1961; 86: 554-555.
2. Gregoir W, Van Regemorter GV. Le reflux vésico-urétéral congénital. Urol Int. 1964; 18: 122-123.
