# Supplementary material for: Frailty predicts surgical complications after kidney transplantation. A propensity score matched study
Source: PLoS One. 2020 Feb 26;15(2):e0229531. doi: 10.1371/journal.pone.0229531 (PMC7043931; doi:10.1371/journal.pone.0229531)
Supplement: S2 Appendix — (PDF) [file pone.0229531.s002.pdf]

Frailty score components[1-3].

<table>
<tr><td>1 - Shrinking:</td><td colspan="4">Self-reported unintentional weight loss in the last year.
Point earned if: ≥ 10 lb lost in the previous year and/or BMI < 18.5 kg/m^2.</td></tr>
<tr><td rowspan="3">2 - Weakness:</td><td colspan="4">Using a hand-held dynamometer in the arm without a functioning shunt, performed in triplicate, requesting the individual to exert the greatest possible force.
Point earned if:</td></tr>
<tr><td colspan="2">MEN</td><td colspan="2">WOMEN</td></tr>
<tr><td>BMI (kg/m^2):
≤ 24.0
24.1-26.0
26.1-28.0
> 28.0</td><td>Strength (kg):
≤ 29.0
≤ 30.0
≤ 30.0
≤ 32.0</td><td>BMI (kg/m^2):
≤ 23.0
23.1-26.0
26.1-29.0
> 29.0</td><td>Strength (kg):
≤ 17.0
≤ 17.3
≤ 18.0
≤ 21.0</td></tr>
<tr><td>3 - Exhaustion:</td><td colspan="4">Two questions from the Center for Epidemiological Studies Depression Scale were used. Item seven: "I felt that everything I did was an effort" and item twenty: "I could not get going".
Point earned if: Answered “yes” to either of the two questions.</td></tr>
<tr><td rowspan="3">4 - Slow walking speed:</td><td colspan="4">The time taken to walk 15 ft in a straight line was assessed in triplicate.
Point earned if:</td></tr>
<tr><td colspan="2">MEN</td><td colspan="2">WOMEN</td></tr>
<tr><td>Height (cm):
≤ 173
> 173</td><td>Time (s):
≥ 7
≥ 6</td><td>Height (cm):
≤ 159
> 159</td><td>Time (s):
≥ 7
≥ 6</td></tr>
<tr><td rowspan="3">5 - Low physical activity:</td><td colspan="4">The Minnesota Leisure Time Physical Activity Questionnaire was used, and the weekly caloric expenditure was calculated for each activity performed.
Point earned if:</td></tr>
<tr><td colspan="2">MEN</td><td colspan="2">WOMEN</td></tr>
<tr><td colspan="2">Weekly caloric expenditure (kcal): < 383</td><td colspan="2">Weekly caloric expenditure (kcal): < 270</td></tr>
</table>

References

1. Fried LP, Tangen CM, Walston J, et al. Frailty in older adults: Evidence for phenotype. *J Gerentol A Biol Sci Med Sci*. 2001;564(3):146-156.

2. Batistoni SS, Neri AL, Cupertino AP. Validity of the Center for Epidemiological Studies Depression Scale among Brazilian elderly. *Rev Saude Publica*. 2007;41(4):598-605.

3. Lustosa LP, Pereira DS, Dias RC, et al. Tradução e adaptação transcultural do Minnesota Leisure Time Activities Questionnaire em idosos. *Geriatr Gerontol*. 2011;5(2):57-65.
