# Supplementary figures and images for: Frailty predicts surgical complications after kidney transplantation. A propensity score matched study
Source: PLoS One. 2020 Feb 26;15(2):e0229531. doi: 10.1371/journal.pone.0229531 (PMC7043931; doi:10.1371/journal.pone.0229531)

## S5 Appendix – Distribution of propensity scores after matching.

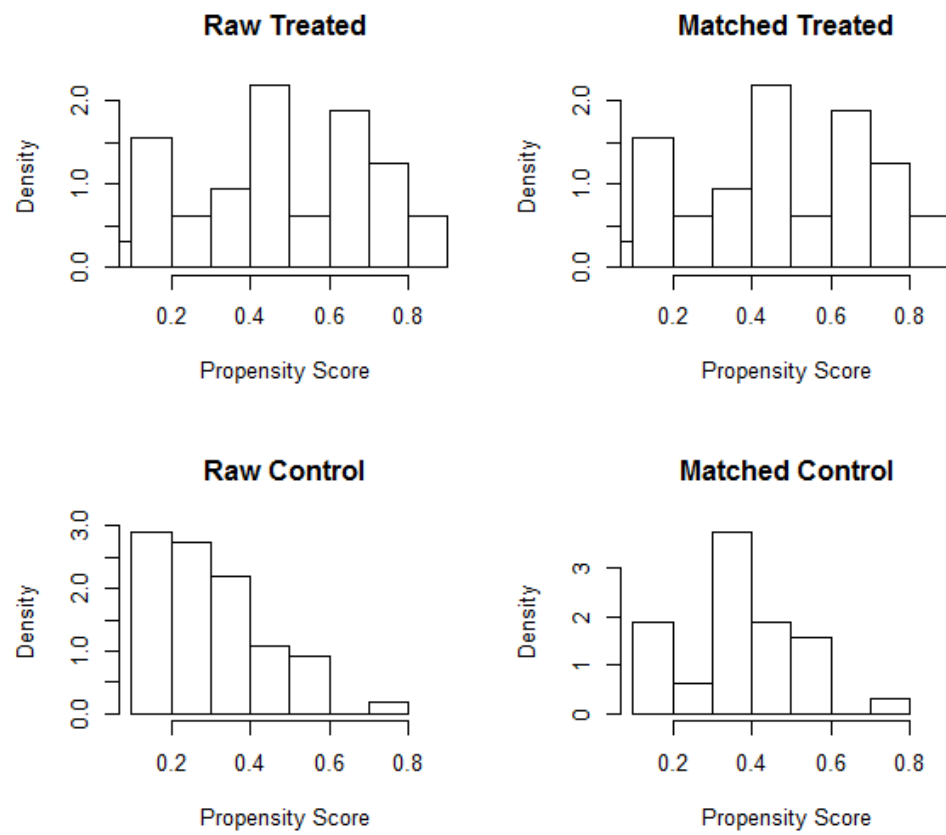

Supplement: S5 Appendix — (PDF) [file pone.0229531.s005.pdf]
