# Supplementary material for: Frailty predicts surgical complications after kidney transplantation. A propensity score matched study
Source: PLoS One. 2020 Feb 26;15(2):e0229531. doi: 10.1371/journal.pone.0229531 (PMC7043931; doi:10.1371/journal.pone.0229531)
Supplement: Attachment — Submitted filename: Response To Reviewers.docx [file pone.0229531.s006.docx]

Dear Editor,

We really appreciate all the comments made about our manuscript. Below we answer the questions and suggestions made by the reviewers.

Dear reviewers,

Thank you very much for the comments. They were extremely helpful. We are available to answer any further questions or suggestions you might have.

Reviewer #1: The manuscript entitled 'Frailty predicts surgical complications after kidney transplantation. A propensity score matched study. ' with the aim to determine of frailty are predictive of surgical complications following kidney transplant.

This is quite an interesting study. The manuscript can be further improved.

Comments

Methods
It would be good to have sample size calculation for the study.

**Answer: We provided the sample size calculation in ‘Statistical analysis’ section.**

Figure 1, more detail information to be provided to describe the study i.e. flow, grouping, M0, M1 etc

**Answer: Done as suggested. Please see the new figure 1 attached.**

Statistical analysis
Statement on statistical analysis were performed on 'before matching' and 'after matching' to be added. 1 or 2 tailed for the statistical test(s) to be stated.

**Answer: Done as suggested.**

Results

Table 1, statistical test to be denoted in the table footnote.

**Answer: Statistical tests were denoted in the Table 1 footnote.**

Line 212- 220, the figure of RR, 95%CI to be placed in Table 3. 95% CI or 95% confidence interval (CI) to be standardized.
**Answer: Confidence interval was standardized in the text. The 95% CI was provided in Table 3.**

Table 3, individual n and total complications for both before and after matching to be added. Statistical test to be denoted in the table footnote.

**Answer:** **Done as suggested.**

Figure 2, 3, n (%) to be labelled in the graph for easy visualization.

**Answer: We modified the figures 2 and 3 for easy visualization.**

Reviewer #2: Predicting complications and hence making the decision as to whether to proceed with transplantation is a complex process. There having been a growing number of publication looking at some for frailty index and this study adds to that body of work and hence is timely. The frailty methodology (Fried) used here is a well validated method of calculating frailty in renal patients.
There are some major questions that need clarification:

1. There were 131 transplants during this period but only 88 were included in the study. The area of concerns is that a total of 24 of the 44 exclusions were attributed to either researcher not informed or logistical difficulties. This needs further to be explained in more detail as there is a real risk of systematic bias.

**Answer: We combined the terms ‘researcher not informed’, ‘logistical difficulties’, and ‘no available time for collection’ into a single term named ‘logistical difficulties’ because they represent similar reasons for non-inclusion in the study. Logistical difficulties occurred mainly due to communication difficulties between the transplantation team and the research team at patient admission. However, we believe that this potential bias does not represent a relevant risk, as it is not possible to identify patients as frail or non-frail without applying the methodology of Fried et al., nor can we anticipate kidney transplant outcomes at the time patients are hospitalized. We added a statement in the ‘Results’ section that explains in more detail the logistical difficulties.**

2. It is stated that iatrogenic surgical complications were excluded from the analysis. It is not clear how it was determined if a complication was iatrogenic. Of note the commonest complication was urinary fistula. Under certain circumstances this could be considered an iatrogenic complication. Similarly it would be useful to know what complications were excluded from consideration as they were thought to be iatrogenic.

**Answer: We have initially proposed to define ‘iatrogenic surgical complications’ as organ and tissue injuries that accidentally occur at the surgical site during the procedure. However, we agree with the reviewer that the definition of ‘iatrogenic surgical complications’ is rather inaccurate. That’s why we removed this statement from the manuscript. Additionally, no patients were excluded due to this condition.**

Reviewer #3: Thank you for the opportunity to review this paper which is of huge relevance and significance within the current transplant climate. I think it has been relatively well written and raises some interesting points.

My main issue with the study is that the measure used to assess frailty does not appear to have been validated for the renal failure population. The measure used (Fried et al) was written "To develop and operationalize a phenotype of frailty in older adults" and was validated based on a sample of patients who were all aged over 65. To use the same measure in the renal failure population awaiting transplantation is problematic for a number of reasons. Firstly, they are, on average, 20 years younger. Secondly, many of the sequela of ESRD are similar to that of frailty and therefore it is impossible to know whether the scores from the questionnaire are demonstrating true frailty or symptoms of ESRD. For example, weight loss is a difficult factor to consider within the dialysis population due to issues with fluid retention, whether patients are on a fluid restriction, whether they had been dialysed prior to admission and so on. It is therefore imperative that a measure of this kind is validated within the population in which you wish to use it.

**Answer: Frailty has been shown to be applicable in end-stage renal disease patients (ESRD) and in kidney-transplanted patients. These populations appear to share many pathogenic mechanisms of frailty, such as a pro-inflammatory state, with an exacerbated production of inflammatory cytokines, and dysregulation of the immune, neuroendocrine, and neuromuscular systems, resulting in accelerated ageing [1-4]. Frailty is considered highly prevalent in patients at any stage of chronic kidney disease (CKD) and may reach up to two-thirds in ESRD cases [5]. The use of the frailty score has progressively increased in the ESRD and transplant population. A recent systematic review [6] evaluating the use of the frailty score in the population of patients with CKD found that 72% of the studies used the Fried et al. score.**

**In kidney transplantation, frailty has been shown to be an independent risk factor for delayed graft function [1], post-transplant delirium [7], longer initial length of hospital stay [8], early hospital readmission [9], adverse effects from immunosuppressive drugs [10], and death [11]. In the ‘Report from the American Society of Transplantation on frailty in solid organ transplantation’, 98.9% of transplant surgeons responded that frailty in transplant candidates is a risk factor for adverse outcomes after transplantation [12].**

**We think that the measure of weight loss when calculating the Fried score in dialysis population was not a significant limitation because we only considered self-report of unintentional dry weight loss, reducing this limitation. Beside that, the Fried score has been used as a robust marker of outcomes in ESRD patients.**

**We added the two following statements in the ‘Introduction’ and ‘Material and methods’ sections, respectively, to emphasize the topics discussed above:**

**- ‘Frailty is considered highly prevalent in patients at any stage of chronic kidney disease (CKD), and may reach up to two-thirds in ESRD cases [Kojima G. Prevalence of frailty in end-stage renal disease: a systematic review and meta-analysis. Int Urol Nephrol. 2017;49 (11):1989–1997]. The use of the frailty score has progressively increased in the ESRD and transplant population. A recent systematic review [Chowdhury R, Peel NM, Krosch M, Hubbard RE. Frailty and chronic kidney disease: A systematic review. Arch Gerontol Geriatr. 2017;68:135–142] evaluating the use of the frailty score in the population of patients with CKD found that 72% of the studies used the Fried et al. score.’**

**- ‘Fried et al. score was calculated considering only the dry weight loss.’**

**References**

1. **Garonzik-Wang JM, Govindan P, Grinnan JW, Liu M, Ali HM, Chakraborty A, et al. Frailty and delayed graft function in kidney transplant recipients. Arch Surg. 2012;147(2):190-193.**
2. **Exterkate L, Slegtenhorst BR, Kelm M, Seyda M, Schuitenmaker JM, Quante M, et al. Frailty and transplantation. Transplantation. 2016;100(4):727-733.**
3. **McAdams-DeMarco MA, Suresh S, Law A, Salter ML, Gimenez LF, Jaar BG, et al. Frailty and falls among adult patients undergoing chronic hemodialysis: A prospective cohort study. BMC Nephrol. 2013;214:224.**
4. **McAdams‐DeMarco MA, Law A, Salter ML, Boyarsky B, Gimenez L, Jaar BG, et al. Frailty as a novel predictor of mortality and hospitalization in individuals of all ages undergoing hemodialysis. J Am Geriatr Soc. 2013;61(6):896-901.**
5. **Kojima G. Prevalence of frailty in end-stage renal disease: a systematic review and meta-analysis. Int Urol Nephrol. 2017;49(11):1989–1997.**
6. **Chowdhury R, Peel NM, Krosch M, Hubbard RE. Frailty and chronic kidney disease: A systematic review. Arch Gerontol Geriatr. 2017;68:135–142.**
7. **Haugen CE, Mountford A, Warsame F, Berkowitz R, Bae S, Thomas AG, et al. Incidence, risk factors, and sequelae of post‐kidney transplant delirium. J Am Soc Nephrol. 2018;29(6):1752-1759.**
8. **McAdams‐DeMarco MA, King EA, Luo X, Haugen C, DiBrito S, Shaffer A, et al. Frailty, length of stay, and mortality in kidney transplant recipients: a national registry and prospective cohort study. Ann Surg. 2016;266(6):1084‐1090.**
9. **McAdams-DeMarco MA, Law A, Salter ML, Chow E, Grams M, Walston J, et al. Frailty and early hospital readmission after kidney transplantation. Am J Transplant. 2013;13(8):2091-2095.**
10. **McAdams-De Marco MA, Law A, Tan J, Delp C, King EA, Orandi, et al. Frailty, mycophenolate reduction, and graft loss in kidney transplant recipients. Transplantation. 2015;15(1):149-154.**
11. **Mc Adams-De Marco MA, Law A, King E, Orandi B, Salter M, Gupta N, et al. Frailty and mortality in Kidney transplant recipients. Am J Transplant. 2015;15(1):149-154.**
12. **Kobashigawa J, Dadhania D, Bhorade S, Adey D, Berger J, Bhat G, et al. Report from the American Society of Transplantation on frailty in solid organ transplantation. Am J Transplant. 2019;19(4): 984-994.**

With regard to the conclusions of the study, I agree that the finding of non-infective complications being at a higher rate in the more frail population is of interest, however I am unsure (given the reasons outlined above) how reliable this data is. Reason being, symptoms or signs that may be labelled as 'frailty' may actually be signs of something else related to the renal failure that would also have put the patient at higher risk of complications.

**Answer: According to the discussion and the literature above, we believe that Fried et al. frailty score is indeed applicable to ESRD and transplant populations. Frailty score can be a robust marker that predicts important outcomes after transplantation. The most important is that the Fried et al. score can capture higher risk patients in kidney transplantation.**

More minor points:
Abstract - I would expand the results and discussion sections. A single line for these parts of the abstract are not really adequate in my opinion

**Answer: We agree with the reviewer. The results and discussion sections in the abstract were expanded.**

I would not class a lymphocele as a urological complication

**Answer: Some references classified the lymphocele as vascular and others as urological complication. We agree to reclassify lymphocele as a vascular complication.**

I am unsure what a urinary fistula is - please explain what is meant by this

**Answer: We refered urinary leak as urinary fistula. We changed the terms in the text.**

Exclusion criteria - you say that 'those whose physical or congnitive conditions prevented the calculation of the frailty score were excluded from the study' however you do not expand on what these are.

**Answer: We changed the phrase to ‘Individuals with amputations or other physical conditions that prevented the walk test or the handgrip strength test and the patients with significant cognitive impairment who were unable to understand and respond to the frailty score questionnaires were also excluded from the study’.**
