# Supplementary material for: Frailty predicts surgical complications after kidney transplantation. A propensity score matched study
Source: PLoS One. 2020 Feb 26;15(2):e0229531. doi: 10.1371/journal.pone.0229531 (PMC7043931; doi:10.1371/journal.pone.0229531)
Supplement: Attachment — Submitted filename: Response To Reviewers v2.docx [file pone.0229531.s007.docx]

Dear Editor,

We sincerely appreciate all valuable comments and suggestions. We have made a great effort to take into account all the suggestions proposed by the reviewers. We are available to answer any further questions or suggestions you might have.

Reviewer #1: Minor comments.

Page 7 Line 161, alfa to be written as alpha.

**Answer: Thank you very much. We changed to “alpha”.**

Page 7 Line 162, to state clearly sample size 56 for whole group or each group. Also to add discussion on power of study.

**Answer: The reviewer is correct. We apologize about the mistake. In fact, we assumed a frequency of surgical complications of 45% in the frail group and 15% in the non-frail group. Considering a power of 0.80, and alpha of 0.05, we calculated a minimum sample size of 68 patients. This information was corrected in the ‘Statistical analysis’ section. We also calculated the *post hoc* power of the study considering the incidence of surgical complications after matching and it reached 0.71. However, it is important to notice that there was a significant difference between the groups, thus type I error and not type II is the most important in our study.**

Page 12 Table 3, CI 95% to be written as 95%CI.

**Answer: Done as suggested.**

Reviewer #2: I raised 2 questions when originally reviewing this paper. The authors have answered question 2 satisfactorily in my opinion. As for question 1, they have partly addressed my concerns but not fully. I think it is worth their including a statement raising the possibility that the exclusion of patients due to logicistical difficulties may have resulted in a systematic bias. Perhaps the clinical team did not try as hard to contact the Research team for a certain "type" of patient. I think a statement addressing this and a comment of why they do not think it relevant would satisfy me. If the editors are happy with the changes made I do not need to see the manuscript again

**Answer: We added the following statement in the ‘Discussion’ section: ‘The exclusion of patients due to logistical difficulties may have resulted in a systematic bias. However, we believe that this potential bias does not represent a relevant risk, as we cannot anticipate the frail condition without applying the Fried score [13]’.**

Reviewer #3: Thank you for providing a detailed response to my comments and for making appropriate adjustments to the manuscipt.

My main comment is with regard to the statement about questionnaire validation. I think it is entirely reasonable to make a statement saying that frailty is being increasingly assessed within this population and that the Fried score appears to be the most popular rating scale being used. However, I still think there also needs to be a statement that says something akin to "The Fried frailty index has not been formally validated within the end-stage renal failure population, however it is currently the most popular / frequently used questionnaire".

This is important because the reader needs to be aware that whilst this is the most popular scale, it has not undergone the formal validation process that a lot of questionnaires should have done when being used in certain populations. The crossover of symptoms between those with frailty due to renal disease and general frailty is significant and the fact that this has not specifically been considered as part of validity testing is hugely relevant within this context.

**Answer: Thank you very much for the comments. In the ‘Introduction’ section we changed the phrase ‘Frailty is a measure of physiological reserve, initially validated for the geriatric population [13]. However, it has been shown to be applicable in end-stage renal disease (ESRD) patients and in kidney-transplanted patients. These populations appear to share many pathogenic mechanisms of frailty, such as a pro-inflammatory state, with an exacerbated production of inflammatory cytokines, and dysregulation of the immune, neuroendocrine, and neuromuscular systems, resulting in accelerated ageing [12,14-16]’ to ‘Frailty is a measure of physiological reserve, initially validated for the geriatric population [13]. Although the frailty score has not been formally validated for patients with end-stage renal disease (ESRD) and for kidney-transplanted patients, it has been shown to be applicable to these populations. These patients appear to share many pathogenic mechanisms of frailty, such as a pro-inflammatory state, with an exacerbated production of inflammatory cytokines, and dysregulation of the immune, neuroendocrine, and neuromuscular systems, resulting in accelerated ageing [12,14-16]’.**

**We think this statement has clarified the fact that frailty score has not yet undergone the formal validation process within ESRD and kidney-transplanted populations.**
